# Treatment of Cirrhosis-Associated Hyponatremia with Midodrine and Octreotide

**DOI:** 10.3389/fmed.2017.00017

**Published:** 2017-03-14

**Authors:** Sharad Patel, Dai-Scott Nguyen, Anjay Rastogi, Minh-Kevin Nguyen, Minhtri K. Nguyen

**Affiliations:** ^1^David Geffen School of Medicine at UCLA, Los Angeles, CA, USA

**Keywords:** sodium, hyponatremia, cirrhosis, midodrine, octreotide

## Abstract

**Background:**

Hyponatremia in the setting of cirrhosis is a common electrolyte disorder with few therapeutic options. The free water retention is due to non-osmotic vasopressin secretion resulting from the cirrhosis-associated splanchnic vasodilatation. Therefore, vasoconstrictive therapy may correct this electrolyte abnormality. The aim of this study was to assess the efficacy of midodrine and octreotide as a therapeutic approach to increasing urinary electrolyte-free water clearance (EFWC) in the correction of cirrhosis-associated hyponatremia.

**Methods:**

This observational study consisted of 10 patients with cirrhosis-associated hyponatremia. Hypovolemia was ruled out as the cause of the hyponatremia with a 48-h albumin challenge (25 g IV q6 h). Patients whose hyponatremia failed to improve with albumin challenge were started on midodrine and octreotide at 10 mg po tid and 100 μg sq tid, respectively, with rapid up-titration as tolerated to respective maximal doses of 15 mg tid and 200 μg tid within the first 24 h. We assessed urinary EFWC and serum sodium concentration before and 72 h after treatment.

**Results:**

Pretreatment serum sodium levels ranged from 119 to 133 mmol/L. The mean pretreatment serum sodium concentration ± SEM was 124 mmol/L ± 1.6 vs 130 mmol/L ± 1.5 posttreatment (*p* = 0.00001). The mean pretreatment urinary EFWC ± SEM was 0.33 L ± 0.07 vs 0.82 L ± 0.11 posttreatment (*p* = 0.0003).

**Conclusion:**

Our data show a statistically significant increase in serum sodium concentration and urinary EFWC with the use of midodrine and octreotide in the treatment of cirrhosis-associated hyponatremia.

## Introduction

Hyponatremia, defined by a serum sodium concentration of <135 mmol/L, is associated with increased morbidity and mortality in patients with cirrhosis (1–3). Approximately 22% of patients with cirrhosis have hyponatremia, and it is considered to be an independent predictive factor for survival in these patients (4, 5). At this time, the treatment of patients with hyponatremia in the setting of cirrhosis is limited to free water restriction due to recent concerns that tolvaptan—a selective vasopressin 2-receptor antagonist commonly used to treat euvolemic and hypervolemic hyponatremia—carries a potential risk of hepatotoxicity (6).

Decreased effective circulating volume secondary to splanchnic vasodilatation plays a central role in the pathogenesis of hyponatremia in patients with cirrhosis. The peripheral vasodilatation hypothesis, which states that systemic vasodilation leads to a state of salt and water avidity, was described by Schrier et al. in 1988 (7). The splanchnic vasodilatation and resultant reduction in systemic vascular resistance that causes hepatorenal syndrome (HRS) also lead to the development of cirrhosis-associated hyponatremia. The decreased effective circulating volume resulting from the decreased systemic vascular resistance leads to the upregulation of the renin–angiotensin–aldosterone system, norepinephrine, and vasopressin. This non-osmotic release of vasopressin leads to the impaired ability of the kidney to excrete electrolyte-free water, thereby resulting in the development of hyponatremia.

Given the mechanism of splanchnic vasodilatation as the underlying pathophysiology of HRS, several studies have assessed the use of vasoconstrictor therapies as treatment of HRS. Midodrine, which is an alpha agonist, and octreotide, a somatostatin analog, have been shown to be effective in improving renal function in patients with HRS (8, 9). We propose that cirrhosis-associated hyponatremia may respond to treatment with vasoconstrictor therapy since the non-osmotic release of vasopressin is due to the underlying splanchnic vasodilatation. Increasing splanchnic vasoconstriction may improve the diminished effective circulating volume, which in turn would reduce vasopressin release, thereby allowing for more urinary electrolyte-free water excretion (10).

In the current study, we treated a small group of patients who had cirrhosis complicated by hyponatremia and ascites with midodrine and octreotide. The main outcome measures were serum sodium concentration and urinary electrolyte-free water clearance (EFWC).

## Materials and Methods

We performed an observational study to evaluate the efficacy of midodrine and octreotide in the treatment of hyponatremia in patients with cirrhosis. Participants in the study were recruited at Ronald Reagan UCLA Medical Center between December 2013 and December 2014. The study subjects were patients who were referred to the renal consult service with cirrhosis-associated hyponatremia. The study was approved by our institutional IRB, and informed consent was obtained from the participating subjects.

The inclusion criteria included patients with previous diagnosis of cirrhosis with ascites (as evidence of portal hypertension), age >18, serum sodium concentration of <135 mmol/L, and whose hyponatremia failed to improve with albumin challenge for 48 h. Patients whose respiratory status precluded withdrawal of diuretics and fluid challenge with albumin infusion for 48 h and patients whose hyponatremia improved with albumin challenge were excluded from the study. In addition, exclusion criteria included requirement for renal replacement therapy, inability to tolerate midodrine/octreotide (e.g., bradycardia, nausea), and a hospital stay less than the study requirement (<72 h after initiation of midodrine and octreotide).

All patients are placed on 1-L fluid restriction, and all patients received albumin infusion (25 g IV q6 h) for 48 h to exclude hypovolemia as the cause of the hyponatremia. Patients whose hyponatremia failed to improve with albumin challenge for 48 h were then started on midodrine (Mylan Institutional Inc., Rockford, IL, USA) and octreotide (Fresenius Kabi, Lake Zurich, IL, USA) at 10 mg po tid and 100 μg sq tid, respectively, with rapid up-titration as tolerated to respective maximal doses of 15 mg tid and 200 μg tid within the first 24 h.

Serum [Na^+^] and urinary [Na^+^] and [K^+^] were measured by ion selective electrode using the Cobas 8000 analyzer. Serum creatinine was measured by the enzymatic method using the Cobas 8000 analyzer. We recorded serum sodium level and serum creatinine before treatment and 72 h after initiation of midodrine and octreotide. Urinary sodium, potassium, and volume were checked in order to calculate urinary EFWC (11). Urinary EFWC was calculated prior to treatment and 72 h after therapy. The primary outcome was the change in serum sodium concentration before and after treatment with midodrine and octreotide. The secondary outcome was the change in urinary EFWC before and after treatment with midodrine and octreotide.

### Statistics

Paired *t*-test (two-tailed) was performed to determine statistical significance. A *p* value of <0.05 was considered statistically significant.

## Results

From December 2013 to December 2014, a total of 10 patients were recruited into the study. All 10 patients were identified as having a previous diagnosis of cirrhosis with ascites (as evidence of portal hypertension), and they were being consulted for evaluation of hyponatremia. Serum sodium levels prior to treatment ranged from 119 to 133 meq/L. The mean serum sodium concentration ± SEM prior to treatment was 124 meq/L ± 1.6 compared to the mean serum sodium concentration of 130 meq/L ± 1.5 posttreatment (*p* = 0.00001). The mean urinary output ± SEM pretreatment was 0.60 L ± 0.13 vs 1.33 L ± 0.14 posttreatment (*p* = 0.0001) (Table 1), and the mean urinary EFWC ± SEM pretreatment was 0.33 L ± 0.07 vs 0.82 L ± 0.11 posttreatment (*p* = 0.0003). However, there was no significant difference in the mean serum creatinine concentration ± SEM pre- and posttreatment (1.37 ± 0.25 vs 1.45 ± 0.28, *p* = 0.32).

**Table 1:** **Patient data**.

Patient	UOP pretreatment (L)	UOP posttreatment (L)	Urine [Na^+^] pretreatment (mmol/L)	Urine [Na^+^] posttreatment (mmol/L)	Urine [K^+^] pretreatment (mmol/L)	Urine [K^+^] posttreatment (mmol/L)	Serum [Na^+^] pretreatment (mmol/L)	Serum [Na^+^] posttreatment (mmol/L)	Serum creat pretreatment (mg/dL)	Serum creat posttreatment (mg/dL)	Electrolyte-free water clearance (EFWC) pretreatment (L/day)	EFWC posttreatment (L/day)
1	0.73	1.83	19	23	32	38	119	128	0.9	0.8	0.46	1.07
2	0.66	1.42	20	20	39	35	124	128	0.7	0.7	0.39	0.89
3	0.2	1.3	36	62	49	30	132	136	2.7	2.7	0.09	0.53
4	0.425	0.5	22	20	41	36	122	126	1.2	1.8	0.24	0.31
5	0.335	0.9	20	73	20	21	133	139	2.8	3.2	0.25	0.36
6	0.38	1.02	20	20	36	16	120	124	1.7	1.7	0.23	0.76
7	0.9	1.85	28	20	16	26	119	125	0.8	0.9	0.61	1.26
8	0.36	1.48	64	23	36	11	126	132	1.3	1.1	0.11	1.15
9	1.6	1.85	21	20	58	40	123	131	0.7	0.7	0.71	1.11
10	0.45	1.1	37	20	46	27	121	131	0.9	0.9	0.18	0.76
Mean	0.60	1.33	28.7	30.1	37.3	28	123.9	130	1.37	1.45	0.33	0.82

*p* value	0.0001		0.862		0.042		0.00001		0.318		0.0003	

Our 10 patients tolerated midodrine and octreotide at doses of 15 mg po tid and octreotide 200 μg sq tid, respectively, without any adverse effects.

## Discussion

This study is the first to evaluate the efficacy of midodrine and octreotide in the correction of cirrhosis-associated hyponatremia. In this study, the use of midodrine and octreotide significantly improved serum sodium concentration and urinary EFWC in our small group of patients with cirrhosis complicated by ascites (as evidence of portal hypertension) and whose hyponatremia failed to improve with albumin challenge for 48 h.

Hyponatremia commonly occurs in patients with cirrhosis complicated by portal hypertension. In cirrhosis, the pathogenesis of hyponatremia is thought to be related to arterial vasodilatation in the splanchnic circulation, which is triggered by portal hypertension. The presumed mechanism is that portal hypertension leads to increased production or activity of vasodilators, mainly nitric oxide, in the splanchnic circulation. The splanchnic vasodilatation in cirrhotic patients results in arterial underfilling, and the resultant effective circulating volume depletion in these patients stimulates the secretion of vasopressin. The increased vasopressin secretion leads to increased urinary water reabsorption in the collecting tubule, which in turn results in decreased ability of the kidney to excrete electrolyte-free water (EFWC). Therefore, midodrine and octreotide can be a potential treatment for cirrhosis-associated hyponatremia by ameliorating the decreased effective circulating volume resulting from the splanchnic vasodilatation (10).

In this study, our aim was to evaluate the efficacy of midodrine and octreotide in the treatment of cirrhosis-associated hyponatremia. The patients who were included in our study were cirrhotic patients with ascites (as evidence of portal hypertension) whose hyponatremia did not improve with albumin challenge (25 g IV q6 h) for 48 h. Volume expansion with 48 h of albumin challenge was conducted to rule out hyponatremia caused by hypovolemia, which often is the case given the frequent combination of lactulose and diuretic therapy in cirrhotic patients. We postulate that if midodrine and octreotide were to be effective in increasing splanchnic vasoconstriction, the improvement in the decreased effective circulating volume will lead to reduced non-osmotic release of vasopressin, thereby resulting in less urinary water reabsorption in the collecting tubule and increased urinary electrolyte-free water excretion (EFWC). The increased urinary electrolyte-free water excretion will in turn lead to the correction of the hyponatremia. Indeed, treatment with midodrine and octreotide in our patients resulted in an increase in serum sodium concentration and urinary EFWC in each patient as compared to pretreatment values. In our study, there was no significant difference in the mean serum creatinine concentration pre- and posttreatment (*p* = 0.32). The lack of significant improvement in serum creatinine concentration in our patients was likely due to the fact that 7 out of the 10 patients had fairly preserved renal function with serum creatinine concentration within the normal reference range in our laboratory (0.6–1.3 mg/dL) and/or the decreased muscle mass and lower rate of creatinine production in our cirrhotic patients.

Currently, fluid restriction is the mainstay of therapy of hyponatremia in cirrhotic patients. However, fluid restriction in patients with cirrhosis-associated hyponatremia is often difficult to achieve due to the low urinary output resulting from the increased vasopressin secretion. Indeed, assuming that insensible water loss and fecal water loss approximate the water content of ingested food and water of oxidation (12), electrolyte-free water intake must be restricted to less than urinary electrolyte-free water excretion (as determined by EFWC) in order to achieve negative free water balance (13). Strict free water restriction would therefore be difficult to adhere to in patients with low urinary EFWC. Hence, alternative approaches should be undertaken to increase urinary EFWC in these cases. However, an increase in dietary sodium intake is not a therapeutic option in cirrhotic patients since it will lead to sodium and fluid retention and worsening ascites and peripheral edema. In addition, tolvaptan, a selective vasopressin 2-receptor antagonist commonly used to treat euvolemic and hypervolemic hyponatremia, is contraindicated in the setting of cirrhosis due to the potential risk of hepatotoxicity (6). Similarly, there is concern with the use of conivaptan (a non-selective V1a and V2 receptor antagonist) since V1a receptor blockade may result in a reduction in blood pressure and may also compromise renal function (14). Given the limited therapeutic options in patients with cirrhosis-associated hyponatremia, our findings suggest that midodrine and octreotide may be effective in improving hyponatremia in these patients by increasing urinary EFWC.

### Limitations

Although our results showed statistically significant improvement in serum sodium concentration and urinary EFWC in 10 patients with cirrhosis-associated hyponatremia, there were significant limitations in our study. Our study was neither randomized nor blinded but rather observational in nature. In addition, our sample size was small. Therefore, larger randomized, controlled clinical trials are required to validate our findings that treatment with midodrine and octreotide can improve cirrhosis-associated hyponatremia.

## Conclusion

Although the morbidity and mortality associated with cirrhosis-associated hyponatremia are well defined, treatment remains limited in the form of free water restriction (5, 16–18). Given the dearth of viable treatment options for cirrhosis-associated hyponatremia, we believe that this study positively supports the hypothesis that vasoconstrictor therapy may help correct hyponatremia in cirrhotic patients with portal hypertension. To date, our study is the first to evaluate the use of midodrine and octreotide in the correction of cirrhosis-associated hyponatremia. The use of midodrine and octreotide significantly improved serum sodium concentration and urinary EFWC in our small group of patients with cirrhosis complicated by hyponatremia and ascites. Given the morbidity associated with cirrhosis-associated hyponatremia and the limited therapeutic options in this patient population, additional randomized, controlled studies are needed to substantiate the effectiveness of this novel therapeutic approach in cirrhosis-associated hyponatremia.

## Ethics Statement

This study was carried out in accordance with the recommendations of UCLA Institutional Review Board with written informed consent from all subjects. All subjects gave written informed consent in accordance with the Declaration of Helsinki. The protocol was approved by the UCLA Institutional Review Board.

## Author Contributions

SP: acquisition and analysis of data and drafting of the manuscript. D-SN: analysis of data and preparation of table. AR: analysis of data. M-KN: analysis of data and preparation of table. MKN (corresponding author): conception and design of research, analysis and interpretation of data, drafting and revision of the manuscript, and approval of final version of the manuscript.

## Conflict of Interest Statement

The authors declare that the research was conducted in the absence of any commercial or financial relationships that could be construed as a potential conflict of interest.
